# Risk Factors for Breast Cancer and Expression of Insulin-Like Growth Factor-2 (IGF-2) in Women with Breast Cancer in Wuhan City, China

**DOI:** 10.1371/journal.pone.0036497

**Published:** 2012-05-25

**Authors:** Jun Qiu, Rong Yang, Yanhua Rao, Yukai Du, Fatch W. Kalembo

**Affiliations:** 1 Department of Maternal and Child Health, Tongji Medical College, Huazhong University of Science and Technology, Wuhan, China; 2 Centre of Wuhan City Maternal and Child Health Care, Qiaokou, China; 3 Center of Jiangxia District Maternal and Child Health Care, Jiangxi, China; Univ of Bradford, United Kingdom

## Abstract

**Purpose:**

The purpose of this study was to explore the risk factors for breast cancer and establish the expression rate of IGF-2 in female patients.

**Methods:**

A case control study with 500 people in case group and 500 people in control group. A self-administered questionnaire was used to investigate risk factors for breast cancer. All cases were interviewed during a household survey. Immune-histochemical method was used to inspect the expression of IGF-2 in different tissues (benign breast lesions, breast cancer and tumor-adjacent tissue).

**Results:**

Multivariate adjusted odds ratios and 95% confidence intervals were calculated using unconditional logistic regression. High body mass index (OR = 1.012,95%CI = 1.008–1.016), working attributes (OR = 1.004, 95%CI = 1.002 = 1.006), long menstrual period (OR = 1.007, 95%CI = 1.005–1.009), high parity OR = 1.003, 95%CI = 1.001–1.005) , frequent artificial abortion (OR = 1.004, 95%CI = 1.001–1.005), family history of cancer (OR = 1.003, 95%CI = 1.000–1.005), period of night shift (OR = 1.003, 95%CI = 1.001–1.006), live in high risk environment (OR = 1.005, 95%CI = 1.002–1.008), and family problems (OR = 1.010, 95%CI = 1.005–1.014) were associated with increased risk for breast cancer. In this study, good sleeping status, positive coping strategies, subjective support, and utility degree of social support were associated with reduced risk for breast cancer (OR = 0.998, 0.997, 0.985, 0.998 respectively; 95%CI = 0.996–1.000, 0.994–1.000, 0.980–0.989, 0.996–1.000, respectively). In benign breast lesions, breast cancer and tumor-adjacent tissue, IGF-2 was mainly expressed in the cytoplasm, but its expression rate was different (p<0.05).

**Conclusions:**

The incidence of breast cancer is a common result of multiple factors. IGF-2 is involved in the development of breast cancer, and its expression varies in different tissues (benign breast lesions, breast cancer and tumor-adjacent tissue).

## Introduction

Breast cancer is the most common cancer among women with an estimated 1.38 million new cases diagnosed in 2008 (23% of all cancers), and ranks second overall (10.9% of all cancers) worldwide [1]. Large differences in rates of the disease exist between countries, with higher rates in developed countries (America and West Europe) and lower rates in developing countries (Asia). The incidence of breast cancer in china is on the rise. The rise is probably due to lifestyle changes in women and lack of awareness programs. It is rapidly becoming the number one cancer in females [2].The risk factors for BC include early age of menarche, delayed menopause, contraceptive use, hormonal replacement therapy, above-average body mass index, exposure to environmental pollutants, smoking, and use of alcohol [3]–[5].

Insulin-like growth factors (IGFs) are associated with the development and progression of breast cancer. IGF-1 and IGF-2 transmit their signals through two paralagous receptor proteins located in the plasma membrane: the type 1 IGF receptor and the insulin receptor (IGF receptors). High circulating IGF-1 concentrations and low blood IGF binding protein concentrations are risk factors for several types of cancer including breast cancer [6]–[8]. Some studies indicate that IGF-2 activates ER-α and ER-β and modulates their translocation to the nucleus, membrane organelles and the mitochondria [9]. The purpose of the study was therefore to explore risk factors for breast cancer development and to identify IGF-2 expression rate in invasive breast cancer in women of Wuhan city.

## Methods

### Subjects

A hospital-based case-control study population was recruited from March 2007 to June 2009 in Wuhan city to investigate risk factors of breast cancer (BC). A total of 1000 women were enrolled in the study. Eligible cases were women aged 23–80 years diagnosed with insitu or invasive breast cancer during the studying period. BC patients enrolled in the study were restricted to women identified in the four major public hospitals in the city. These hospitals were Tongji hospital, Union hospital, Hubei Province Cancer Hospital and Wuhan Central Hospital. All cases were histologically confirmed. Upon histological confirmation of diagnosis, only those cases without previous treatment for BC were included in the study. The tumor stage was determined according to the TNM system [10]. Among the 505 eligible cases identified during the study period, 3 refused to participate, 2 died before the interview. Majority of breast cancers were in premenopausal stage, and the most common type was IDC (infiltrating ductal carcinoma).

Controls were women from the general population without breast cancer and mental illness, who were matched by age (±3-years) to an equal number of cases. Controls were randomly selected from the general female population using the Wuhan Resident Registry. Among the 554 eligible controls identified, 39 refused to participate, 15 could not be contacted for interviews. All study participants had to be residents of the Wuhan city for at least 5years.

All 1000 women in the study completed a self-administered questionnaire. Information on demography, smoking, use of alcohol, sleeping status, living environment, reproductive history and other breast cancer risk factors was collected. Interviews included household survey and hospitals interviews. All participants were interviewed with LES, TCSQ, and SSRS.

In order to establish the expression of IGF-2, we selected 63 new BC cases in 500 breast cancer cases. The cases were selected in 2009, and they had not received any treatment such as chemotherapy or radiation before surgery. Among the 63 cases, 52 cases had infiltrating ductal carcinoma (IDC), 8 cases had duct cell carcinoma insitu (DCIS), and 3 cases had infiltrating lobular carcinoma (ILC). We analysed the expression rate of IGF2 in the three groups of tissues. The groups were; (1) Sixty three pathological samples of invasive breast cancer derived from case group. (2) Matched adjacent normal tissue 5 cm away from the tumors and (3) 70 breast benign lesions derived from the case group.

### Ethical consideration

The study was approved by Wuhan Public Health Ethical Committee. Permission to conduct the study was also obtained from the Administrative council of the four hospitals, where the study subjects were enrolled. Further consent was obtained from the Management of Wuhan City Council. Informed written consent was also obtained from all participants of the study.

### Immuno-histochemical (IHC) method

Anti-IGF2 antibody (H-103) was commercially available from Santa Cruz Company, (Rabbit, and #sc-5622). Slides were deparaffinized using xylene and graded ethyl alcohols and then rinsed in water. After 3%hydrogen peroxidase block for 30 minutes, antigen retrieval was performed by boiling slides in antigen retrieval solution in a microwave oven at half maximum power for 4 min *6times, followed by a 30-minutes cool-down and rinsing in wash buffer. Slides were then sequentially treated with the following reagents in a humidified chamber at room temperature: 10% normal goat serum for 30 minutes, anti-IGF2 antibody overnight, secondary antibody for 30 minutes, signal amplification and chromogen development for 30 minutes each (wash buffer steps were included between each step). Stained slides were then counterstained with hematoxylin. Each run included appropriate controls.

### Statistical analysis

Demographic characteristics were contrasted by case-control status. Characteristics of the participating cases and controls were compared by using t test for continuous variables, and 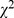 test for continuous variables. Unconditional logistic regression was used to estimate OR (odd ratios) and 95% CI (95% confidence intervals) for the association of life style and breast cancer incidence.

These factors were evaluated by unconditional regression model in order to find the association between them and the risk of breast cancer development. All statistical tests were two-sided. P value less than 0.05 was considered statistically significant. The SPSS version 18.0 statistical software package was used to analyze the data.

## Results

### Sociodemographic characteristics and reproductive variables

Table 1 shows the frequency distributions of sociodemographic and study variables for cases and controls. As a result of frequency matching, cases and controls were similar with respect to likelihood of age and educational levels (*p*>0.05 for all comparisons). High BMI was associated with increased incidence of breast cancer (*p*<0.05). Only 32.9% women chose breast feeding for their children in cases as compared to 54.4% in controls. The difference was statistically significant (*P*<0.05). Controls had late age of menarche, younger age at first live birth, a greater percentage of breastfeeding, high parity, a high frequency of abortion and were less likely to have a personal history of breast fibroadenoma than cases. The difference of two groups was statistically significant (*p*<0.001).

**Table 1:** Sociodemographic characteristics and reproductive health variables among cases and Controls.

	Cases(n = 500)		Controls(n = 500)		P value
	n	%	n	%	
Age					
≤30	12	2.4	14	2.8	
31∼40	68	13.6	65	13.0	
41∼50	201	40.2	196	39.2	
51∼60	153	30.6	158	31.6	
61∼70	40	8.0	38	7.6	
≥71	26	5.2	29	5.8	
Educational level					0.977
Elementary school or below	88	17.4	91	18.2	
Junior high school	169	33.8	159	31.8	
Senior high school	116	23.2	119	23.8	
Junior college	71	14.2	73	14.6	
College or above	56	11.2	58	11.6	
Body mass index					<0.0001
<18.5	23	4.6	26	5.2	
18.5∼22.9	217	43.4	392	78.4	
23∼24.9	209	41.8	76	15.2	
>25	51	10.2	6	1.2	
Age of menarche					
≤12	162	32.4	78	15.6	<0.0001
12∼14	219	43.8	223	44.6	
≥15	119	23.8	199	39.8	
Parity					<0.001
nulliparous	16	3.2	17	3.4	
1	231	46.2	151	30.2	
2	224	44.8	253	50.6	
3	26	5.2	61	12.2	
4^+^	3	0.6	18	3.6	
Age at first live birth years					<0.001
≤20	14	2.8	13	2.7	
21∼24	19	3.9	29	6.0	
25∼29	193	39.9	215	44.5	
30∼34	208	43.0	221	45.7	
≥35	50	10.4	5	1.1	
Number of abortion					<0.001
0	74	14.8	98	19.6	
1	131	26.2	276	55.2	
2	224	44.8	82	16.4	
3	46	9.2	34	16.8	
4^+^	25	5.0	10	2.0	
Lactation situation					<0.001
Breast feeding	159	32.9	263	54.4	
Mixed feeding	248	51.2	170	35.2	
Artificial feeding	77	15.9	50	10.4	
Benign breast disease					<0.001
Yes	344	68.8	57	11.4	
No	156	31.2	443	88.6	

### Cancer risk factors

Step conditional logistic regression showed that high BMI, working conditions, delayed menopause, high parity, increased number of induced abortion, family history of breast cancer, many years of night shift work, living environment with high-risk factors, family problems, and a higher total score of social life events were associated with increased likelihood of breast cancer development (*P*<0.05). Good sleeping behaviour, positive coping strategies, and higher score of subjective support and utility degree of social support were associated with decreased likelihood of breast cancer development (*P*<0.05) (Table 2).

**Table 2:** Multiple conditional logistic regression analysis for the risk factors of breast cancer.

Factors	β	Standard error	OR	95%CI	χ^2^	*P*
Body mass index	0.0120	0.00187	1.012	1.008–1.016	23.8670	<.0001
Working attributes	0.00386	0.000998	1.004	1.002–1.006	7.3274	0.0068
Number of days for menses	0.00672	0.00107	1.007	1.005–1.009	28.4188	<.0001
Parity	0.00268	0.000997	1.003	1.001–1.005	8.5068	0.0035
Number of abortions	0.00309	0.00118	1.004	1.001–1.005	8.7991	0.0030
Family history of BC	0.00265	0.00136	1.003	1.000–1.005	5.5983	0.0180
Sleeping status	−0.00207	0.00104	0.998	0.996–1.000	4.9675	0.0258
Period of night shifts	0.00342	0.00128	1.003	1.001–1.006	3.9365	0.0472
Living environment	0.00478	0.00153	1.005	1.002–1.008	8.5892	0.0034
Positive coping	−0.00273	0.00163	0.997	0.994–1.000	3.5443	0.0598
Subjective support	−0.0156	0.00222	0.985	0.980–0.989	6.0298	0.0141
Utility degree of social support	−0.00207	0.000872	0.998	0.996–1.000	6.8619	0.0088
Family problems	0.00952	0.00223	1.010	1.005–1.014	14.1553	0.0002

### Clinical foundation data in IHC experiment

#### Age

63 cases and 70 controls were included in the immune-histochemical experiment and their ages were well matched (age/yr:49.1±9.76 in cases *versus* 49.0±10.09 in controls, t = 0.07, *P* = 0.943). Maximum age and minimum age in both groups were the same. The oldest was 71 years old, and the youngest was 25 years old.

#### Post-menopausal women

In invasive breast cancer group, there were 42(66.7%) post-menopausal cases. While in benign breast lesions group there were 47 (67.1%) post-menopausal cases. There was no statistical difference between the two groups of subjects in terms menopausal status (P = 0.9535) (Table 3).

**Table 3:** Comparison of age and menopausal status in invasive breast cancer group and benign breast group.

		Invasive BC group	Benign breast group	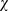 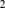	*P*
Age	≤40 yr	11(17.5%)	13(18.6%)	0.1725	0.9965
	41–45 yr	15(23.8%)	15(21.4%)		
	46–50 yr	13(20.6%)	15(21.4%)		
	51–55 yr	9(14.3%)	11(15.7%)		
	≥56 yr	15(23.8%)	16(22.9%)		
Postmenopausal state	postmenopausal	42(66.7%)	47(67.1%)	0.0034	0.9535
	premenopausal	21(33.3%)	23(32.9%)		

#### Body mass index (BMI)

In the 63 cases with invasive breast cancer, the mean body mass index was 22.7±2.65 (kg/m^2^). The maximum (BMI) was 29.7 kg/m^2^, and the minimum was 16.0 kg/m^2^. In the 70 cases with breast benign lesion, the mean body mass index was 21.4±2.09 (kg/m^2^). The maximum (was 27.8 kg/m^2^, and the minimum was 16.6 kg/m^2^. The difference of two groups was statistically significant (*t* = 3.13, *P* = 0.0022) (data not included in the tables).

#### Histological grades

According to the standard of histological grade, 21 cases were classified into grade I, 34 into grade II, and 8 cases into grade III. There were 25 cases without the metastasis of lymph node in the invasive breast cancer. However, there were also 38 cases with the metastasis of lymph node and other tissues. In 63 cases of invasive breast cancer, 41 cases had tumors that were more or equal to 3 cm in size, the remaining 22 cases the tumors were less than 3 cm. Estrogen receptor was found positive in 33 cases. In benign breast lesions patients, 57 cases had tumors less than 3 cm (data not included in the tables).

#### IGF-2 expression

**Figure 1:**
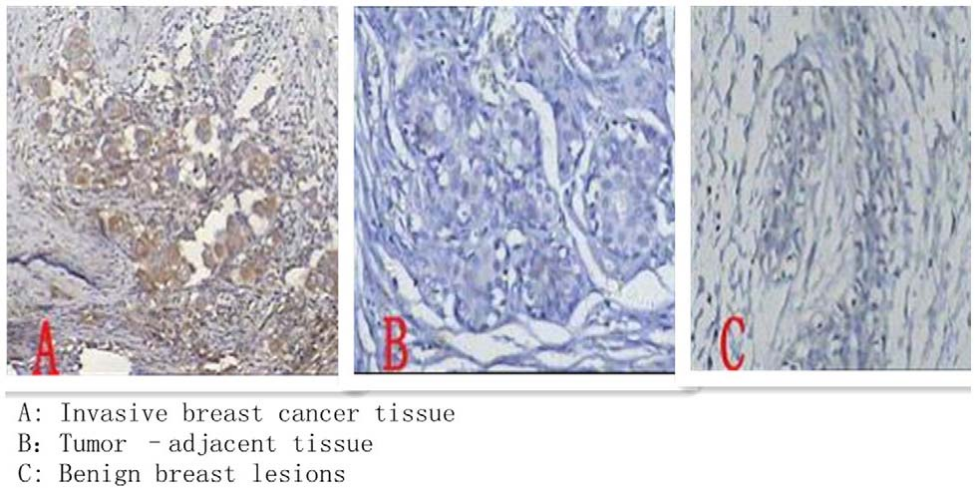
Shows the expression of IGF-2 in different tissues. Figure 1A : Shows the expression of IGF-2 in the invasive breast cancer tissue. Figure 1B: Shows the expression of IGF-2 in the tumor –adjacent tissue. Figure 1C : Shows the expression of IGF-2 in the benign breast lesion tissue.

In benign breast lesions, breast cancer and tumor-adjacent tissue, IGF-2 was mainly expressed in the cytoplasm, but in different tissues the expression was different (p<0.05).In 63 cases of breast invasive ductal cancer, 26 cases were positive for IGF-2.While 7 of 59 cases of breast cancer-adjacent tissue, the expression of IGF -2 was positive, but most of them were weakly positive. In 17 of 70 cases of benign breast lesion patients, IGF-2 was positive but its expression rate was lower than in invasive breast cancer. (See Fig. 1). The expression of IGF- 2 in three types of tissue was significant (*P* = 0.0001). The expression level of IGF -2 in invasive ductal carcinoma was higher than in tumor-adjacent tissue and the difference was statistically significant (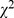 = 13.35, P = 0.0003). In benign breast lesions, however, the expression rate of IGF 2 was not statistically different from breast cancer and the adjacent tissues (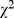 = 4.37 and 3.26 respectively; *P* = 0.0365 and 0.0709 respectively). (Table 4)

**Table 4:** The comparison of IGF-2 expression in breast cancer and the adjacent tissues.

Types of tissue	IGF2 expression		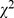	*P*
	Negative N (%)	Positive N (%)		
Breast cancer tissue	37(58.7)	26(41.3)	13.35*	0.0003
Tumor-adjacent tissue	52(88.1)	7(11.9)	4.37▵	0.0365
Benign breast lesions	53(75.7)	17(24.3)	3.26▴	0.0709
Total	89(73.0)	33(27.0)		

* Comparison between the first group and the second group.

▵ Comparison between the first group and the third group.

▴ Comparison between the second group and the third group.

The expression of IGF- 2 in breast cancer was not related to patients' age, menopause or tumor size. In cases with different ages, menopausal status and different sizes of primary lesion, the expression of IGF-2 was not statistically different (P>0.05. A significant relationship was observed, however, among breast cancer with different histological grade, with or without lymph node metastasis and with or without oestrogen receptor positivity in the expression of IGF-2, and the difference was statistically significant (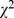 = 14.17, 5.10, 7.60 respectively; *P* values 0.0002, 0.0239, 0.0058 respectively). Rank correlation analysis showed that the expression of IGF-2 in breast cancer tissue was positively correlated to tumor histological grade, lymph node metastasis and the expression of oestrogen receptor (Table 5).

**Table 5:** Expression of insulin-like growth factor 2 (IGF-2) in breast cancer.

Group		Total case	IGF2positive	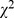	*P*
Age(median = 49 years)	≦49years	32	14(43.8%)	0.17	0.6846
	>49years	31	12(38.7%)		
Menopausal	Postmenopausal	42	17(40.5%)	0.03	0.8564
	premenopausal	21	9(42.9%)		
Tumor size	≤3centimetre	41	15(36.6%)	1.06	0.3025
	>3centimetre	22	11(50.0%)		
Histological grade	1	21	2(9.5%)	14.17*	0.0002
	2	34	18(52.9%)		
	3	8	6(75.0%)		
Lymph node metastasis	yes	38	20(52.6%)	5.10#	0.0239
	no	25	6(24.0%)		
ER	positive	33	19(57.6%)	7.60⊿	0.0058
	negative	30	7(23.3%)		

* r_s_ = 0.4756, P<0.0001;

# r_s_ = 0.2845, P = 0.0238;

⊿ r_s_ = 0.3474, P = 0.005.

## Discussion

BC is a major public health problem affecting millions of women worldwide. With industrialization and urban development, delayed or reduced fertility, increased longevity and altered lifestyle, the incidence of BC is rising steadily even in developing countries. Analysis of data showed that most breast cancer patients were between 40 and 60 years old. The reason behind this is gradual decrease in ovarian function which is common during this period and increase in anterior pituitary activities which result in more estrogen being produced by adrenal cortex, and consequently excessive proliferation of mammary gland epithelial cells. It is therefore important for health workers to encourage women who are more than 30 years old to have regular breast examination once a year.

The results of the study show that the risk of breast cancer development increases significantly with increasing level of body mass index (BMI). The association is clear between high BMI and the increasing Insulin and IGFs. Insulin promotes cancer cell growth, IGFs stimulate cell turnover in most body tissues. [11]–[12]. Significant epidemiological studies suggest that high fat intake, defined as a high BMI increases circulating oestrogens levels [13]–[14]. It is therefore important to advise women to follow good eating habits by eating nutritious food which is low in fats and to do regular body exercises in order to prevent obesity.

The relationship between breast cancer and occupation is under discussion. Occupation as analysed by the multivariate conditional logistic regression in this research revealed that the difference in work pressure and working conditions between various occupations can explain part of this phenomenon. A related study also found an association between night shift work and breast cancer risk among women [15]. In a large prospective cohort study which included 78,562 women from the Nurses' Health Study, found an increased incidence of breast cancer among postmenopausal women who had worked for 30 or more years on rotating night shifts (RR = 1.36; 95% CI = 1.04–1.78) [16]. It is important for Policy makers to ensure that work policies are made or amended to limit the number of night shifts work for women.

Numerous studies confirm that a small age of menarche and more years of menstruation are associated with high risk for breast cancer development. Literature also indicates that women whose menstrual age is more than 40 years have a double risk for breast cancer development as compared to women whose menstrual age is less than 30 years [17]. According to a previous research, early abortion was found as a risk factor for breast cancer development. In this study, menstrual period, parity and number induced abortion were analysed by multivariate logistic regression model, which revealed that they are risk factors for breast cancer. Therefore, pregnancy during marriage should be recommended and unwanted pregnancy should be prevented by encouraging women to use family planning methods. Those who have had induced abortion should be screened regularly and encouraged to do self-breast examination.

Analysis of data by logistic regression model in this study showed that lifestyle changes can have an effect on the risk of developing breast cancer; this is also supported by several lines of evidence in this research. Good quality of sleep is a protective factor, while longer working period of night shift in a year was entered in logistic regression mode as a risk factor. The body produces melatonin during night, which protects deoxyribonucleic acid (DNA) from being damaged by oxide in the body; simultaneously it can inhibit the production of estrogen. Long-term night shift will disrupt the rhythm of life, and then will increase the level of circulating estrogen, consequently inhibiting the body's tumor suppressor mechanism [18].

It can therefore be seen that the more women take negative response to breast cancer risk factors, the more susceptible they are to breast cancer. Subjective support and the high use of social support reduce the incidence of breast cancer [15]. Metastatic breast cancer patients with low cortisol concentrations may take better social support. In other words, they have more healthy neuroendocrine function. A previous study also showed that breast cancer is related to some life events [19].

Insulin-like growth factors system (IGFs) is composed of three ligands, namely, IGF-1, IGF-2, and insulin, which transmit their signals through two paralagous receptor proteins located in the plasma membrane: the type I IGF receptor and the insulin receptor (IGF receptors). The IGF system is involved in tumorigenesis and the proliferation, survival, and migration of tumor cells. High circulating IGF-1 concentrations and low blood IGF binding protein concentrations are a risk factor for several types of cancer including breast cancer [6], [20]–[21]. Elevated circulating IGF-1 levels have been significantly associated with an increased risk of breast cancer, while the evidence for IGF-2 is less clear. IGF2, a growth-promoting, mitogenic and anti-apoptotic factor, plays a key role in the initiation and progression of several cancers [22]. The IGF-2, an imprinted gene with paternal allele expressed and maternal allele silenced, is an important autocrine growth factor in tumors due to its mitogenic and antiapoptotic functions mediated by receptor, which is suggestive of its role in the development of breast cancer [23]. A number of studies have shown consistently the loss of imprint of IGF-2, which are associated with increased risk of several cancers, including those cutaneous melanoma, laryngeal squamous cell carcinoma, human meningiomas and breast cancer [24]–[26]. Evidence from epidemiology studies on the association of IGFs and risk for cancer development has been indicating a potential role of IGFs in breast carcinogenesis [27]. A recent study claims that loss of imprinting (LOI) of IGF-2 gene defines a molecular subgroup of Wilms tumors that have a different pathologic subtype, a later age of onset, and greater IGF-2 expression than those without LOI [28].

In our study, Among the 63 cases of breast invasive ductal cancer patients, 26 cases were positive for IGF2, and the rate of IGF-2 gene expression in the invasive breast cancer was higher than in the tumor-adjacent tissue. This observation supports the findings of studies which also showed an increased expression of IGF2 protein by IHC in BC [29]–[30]. The rate of IGF-2 gene expression in cases with lymph node metastasis was higher than in patients without lymph node metastasis. The grade of tumor pathology was higher, while the rate of IGF-2 gene expression was also higher. The difference in different grades was statistically significant. The outcomes have shown that IGF-2 may play important role in the invasive growth and evolution of tumor. The IGF-2 can promote the synthesis of calcium sticky protein, fiber connection protein, laminnin and other adhesion molecules of which their functions can increase its endothelium basement membrane adhesion. The insulin-like growth factor-II(IGF-2) is a potent mitogen that plays an essential role not only in normal growth and development, but also in breast cancer susceptibility, growth and progression by signalling the IGF1 and insulin receptors [31]–[35] .The proliferation of breast cancer cells in culture is also responsive to insulin-like growth factors, and components of the IGF-1signal transduction system are expressed by both breast tumors and cultured breast cancer cells. Based on this explanation, it can be concluded that the expression of IGF-2 gene in invasive BC is related to the transfer of tumor.

In this study we observed a positive association between IGF-2 and the estrogen receptor. Recently, insulin and IGF have been associated with regulation of sex hormone binding globulin, which modifies the availability of estrogen [36]. Estradiol increased insulin receptor substrate-1 mRNA and protein levels at concentrations consistent with a mechanism involving the estrogen receptor. Almost all members of the IGF system are regulated by the transcriptional level of ER. At the same time, ER can regulate the transcription of IGF1, IGF2, IGF-1R, and IGF-BP [37]. In the study, the results showed that the expression of IGF-2 in ER-positive BC tissues was significantly higher than in ER-negative BC tissues. This further confirms that the expression of IGF-2 positively correlated to the expression of ER in BC tissue. The study found that the expression of IGF-2 was related to histological grade, lymph node metastasis and ER status. Therefore, it implies that IGF-2 may have certain significance for the treatment and prognosis of breast cancer.

The following limitations might have affected the results of our study. Firstly, inaccuracy in estimating BMI. Data on weight and height, which were used to calculate BMI, were based on self-reports and might not reflect the true values. Another potential limitation is recall bias, it is possible that our subjects underreported or over reported some information in their response to the questionnaire however, if recall bias was present in our study population, we believe that it acted similarly among cases and controls.

### Conclusion

The incidence of breast cancer is a result of multiple factors, such as BMI, vocation, menstruation disorder, family history of breast cancer, lifestyle and other pyscho-social factors. IGF-2 is involved in the development of breast cancer, and its expression rate is not the same in different tissues. It is therefore important for women, to reduce negative life events and negative emotional responses. Women should be encouraged to increase the use of social support in order to antagonize the negative impact of life events that can predispose them to breast cancer. Further studies are required to explore the role of IGF-2 in breast cancer development and progression. Health workers should encourage women to comply with recommended breast cancer screening so that exposure to risk factors is detected treated in time.

## References

[1] Munagala R, Aqil F, Gupta RC (2010). Promising molecular targeted therapies in breast cancer.. Indian Journal of Pharmacology.

[2] Ferlay J, Shin FJ, Bray F, Forman D, Mathers C (2010). Estimate of worldwide burden of cancer in 2008.. Int J Cancer.

[3] Hulka BS, Moorman PG (2001). Breast cancer: hormones and other risk factors.. Maturitas.

[4] Kristensen VN, Borresen-Dale AL (2000). Molecular epidemiology of breast cancer: genetic variation in steroid hormone metabolism.. Mutation Research.

[5] Kang HJ, Kim SW, Ahn SJ, Bae JY, Park SK (2002). Polymorphisms in the estrogen receptor-alpha gene and breast cancer risk.. Cancer Letters.

[6] Hankinson SE, Willett WC, Colditz GA, Hunter DJ, Michaud DS (1998). Circulating concentrations of insulin like growth factor-I and risk of breast cancer.. Lancet.

[7] Schernhammer ES, Holly JM, Pollak MN, Hankinson SE (2005). Circulating levels of insulin-like growth factors, their binding proteins, and breast cancer risk.. Cancer Epidemiol Biomarkers Prev.

[8] Renehan AG, Harvie M, Howell A (2006). Insulin-like growth factor (IGF)-I, IGF binding protein-3, and breast cancer risk: eight years on.. Endocr Relat.

[9] Richardson AE, Hamilton N, Davis W, Brito C, De León D (2011). Insulin-like Growth Factor-2 (IGF-2) Activates Estrogen Receptor-α and -β via the IGF-1 and the Insulin Receptors in Breast Cancer Cells.. Growth Factors.

[10] American Joint Committee on Cancer: Breast (1980). Handbook for Staging of Cancer.

[11] Wolk A, Gridley G, Svensson M, Nyren McLaughlin JK (2001). A prospective study of obesity and cancer risk.. Cancer Causes Control.

[12] Huang Z, Hankinson SE, Colditz GA, Stampfer MJ, Hunter DJ (1997). Dual effects of weight and weight gain on breast cancer risk.. JAMA.

[13] Harvie M, Howell A, Vierkant RA, Kumar N, Cerhan JR (2005). Association of gain and loss of weight before and after menopause with risk of postmenopausal breast cancer in the Iowa women's health study.. Cancer Epidemiol Biomarkers Prev.

[14] Prentice RL, Caan B, Chlebowski RT, Patterson R, Kuller LH (2006). Low-fat dietary pattern and risk of invasive breast cancer: the Women's Health Initiative Randomized Controlled Dietary Modification Trial.. JAMA.

[15] Lie JA, Roessink J, Kjærheim K (2006). Breast cancer and night work among Norwegian nurses.. J Cancer Causes and Control.

[16] Schernhammer ES, Laden F, Speizer FE, Willett WC, Hunter DJ (2001). Rotating night shifts and risk of breast cancer in women participating in the nurses' health study.. J Natl Cancer Inst.

[17] Molloy CA, May FE, Westley BR (2000). Insulin receptor substrate expression is regulated by estrogen in the MCF27 Human breast cancer cell line.. J Bio Chem.

[18] Kotsopoulos J, Lubinski J, Lynch HT, Neuhausen SL, Ghadirian P (2005). Age at menarche and the risk of breast cancer in BRCA1 and BRCA2 mutation carriers.. J Cancer Causes and Control.

[19] Turner-Cobb JM, Sandra E, Sephton SE, Koopman C, Blake-Mortimer J (2000). Social Support and Salivary Cortisol in Women with Metastatic Breast Cancer.. Journl of Psychosomatic Medicine.

[20] Schernhammer ES, Holly JM, Pollak MN, Hankinson SE (2005). Circulating levels of insulin-like growth factors, their binding proteins, and breast cancer risk.. Cancer Epidemiol Biomarkers Prev.

[21] Renehan AG, Harvie M, Howell A (2006). Insulin-like growth factor (IGF)-I, IGF binding protein-3, and breast cancer risk: eight years on.. Endocr Relat Cancer.

[22] Rosen N, Yee D, Lippman ME, Paik S, Cullen KJ (1991). Insulin-like growth factors in human breast cancer.. Breast Cancer Res Treat.

[23] Renehan AG, Harvie M, Howell A (2006). Insulin-like growth factor (IGF)-I, IGF binding protein-3, and breast cancer risk.. EndocrRelat Cancer.

[24] Soares MR, Huber J, Rios AF (2010). Investigation of IGF2/ApaI and H19/RsaI polymorphisms in patients with cutaneous melanoma.. Growth Hormone & IGF Research.

[25] Grbeša I, Ivkić M, Pegan B, Gall-Trošelj K (2006). Loss of imprinting and promoter usage of the IGF2 in laryngeal squamous cell carcinoma.. Cancer Letters.

[26] Muller S, Zirkel D, Westphal M, Zumkeller W (2000). Genomic imprinting of IGF2 and H19 in human meningiomas.. European Journal of Cancer.

[27] Hankinson SE, Schernhammer ES (2003). Insulin-like growth factor and breast cancer risk: evidence from observational studies.. Breast Diseases.

[28] Ravenel JD, Broman WK (2001). Loss of imprinting of insulin-like growth factor-II (IGF2) gene in distinguishing specific biologic subtypes of wilms tumor.. Journal of the National Cancer Institute.

[29] Giani C, Campani D, Rasmussen A, Fierabracci P, Miccoli P (2002). Insulin-like growth factor II (IGF-II). Immunohistochemistry in breast cancer: relationship with the most important morphological and biochemical prognostic parameters.. Int J Biolog Mark.

[30] Shetty PJ, Movva S, Pasupuleti N (2011). Regulation of IGF2 transcript and protein expression by altered methylation in breast cancer.. J Cancer Res Clin Oncol.

[31] De Myets P (1994). The structural basis of insulin and insulin-like growth factor-1 receptor binding and negative cooperativity, and its relevance to mitogenic versus metabolic signalling.. Diabetologia.

[32] Morrione A, Valentinis B, Xu SQ, Yumet G, Louvi A (1997). IGF-II stimulates cell proliferation through the insulin receptor, Proc.. Natl Acad Sc.

[33] Sciacca L, Costantino A, Pandini G, Mineo R, Frasca F (1999). Insulin receptor activation by IGF-II in breast cancers: evidence for a new autocrine/paracrine mechanism.. Oncogene.

[34] Visual D, Bosma A, Vrieling A, Rookus M, Veer L (2004). IGF system mRNA quantities in normal and tumor breast tissue of women with sporadic and familial breast cancer risk.. Breast Cancer Res Treat.

[35] Pacher M, Seewald MJ, Mikula M, Oehler S, Mogg M (2007). Impact of constitutive IGF1/IGF2 stimulation on the transcriptional program of human breast cancer cells.. Carcinogenesis.

[36] Kaaks R (2001). Plasma insuline, IGF-I et cancer du sein Plasma insulin, IGF-I and breast cancer.. Gynécol Obstét Ferti.

[37] LeRioth D, Roberts CT (2003). The insulin2 like growth factor system and cancer.. Cancer Lett.

